# The importance of productive patient–professional interaction for the well-being of chronically ill patients

**DOI:** 10.1007/s11136-014-0813-6

**Published:** 2014-09-30

**Authors:** Jane Murray Cramm, Anna Petra Nieboer

**Affiliations:** Department of Health Policy and Management (iBMG), Erasmus University Rotterdam, Burgemeester Oudlaan 50, 3062 PA Rotterdam, The Netherlands

**Keywords:** Chronic care, Disease management, Quality of care, Interaction, Well-being, Coordination, Patient-centered

## Abstract

**Objective:**

To investigate patient–professional interactions and identify the association between quality of care, productivity of patient–professional interaction, and chronically ill patients’ well-being.

**Methods:**

Questionnaires were distributed to chronically ill patients [T1 (2011), 2,191/4,693 (47 %) respondents; T2 (2012), 1,722/4,350 (40 %) respondents].

**Results:**

Patients perceived a higher degree of productive interaction with general practitioners compared to other professionals. Bivariate analyses showed that patients’ well-being at T2 was positively related to well-being at T1 (*r* = 0.70), quality of care (*r* = 0.12), and productive patient–professional interaction (*r* = 0.31; all *p* ≤ 0.001). Single status (*r* = –0.14), low education (*r* = –0.11), and female gender (*r* = –0.11; all *p* ≤ 0.001) were negatively associated with well-being. Multivariate analyses showed that after controlling for background characteristics and well-being at baseline quality of care is associated with patients’ well-being at T2 (*p* ≤ 0.01). When productive patient–professional interactions were entered into the equation, they not only were related to patients’ well-being (*p* ≤ 0.001) but also mediated the relationship between the quality of care and well-being. More productive patient–professional interactions were related to better well-being at T2 (*B* = 0.11), assuming that all other factors in the model remained constant.

**Conclusions:**

Productive patient–professional interactions are associated with chronically ill patients’ well-being over time and mediate the relationship between well-being and quality of care. Improvement of the quality of chronic care delivery should always be accompanied by investment in the quality of relationships and communication between patients and professionals.

## Introduction

High-quality chronic care delivery and productive interaction between patients and healthcare professionals are expected to lead to better patient outcomes [1, 2]. Wagner et al. [1–3] developed the chronic care model to guide quality improvement and provide high-quality care delivery through productive interactions with patients. Important elements of the model include strengthening the patient–professional relationship through self-management support, effective use of community resources, integrated decision support for professionals, and the use of patient registries and other supportive information technology [2–4]. Evidence showed that successful improvement strategies in chronic disease care are consistent with the concept of the chronic care model [5] and that professional perceptions of high quality of chronic care delivery predict more positive experiences among chronically ill patients [6]. Current care systems are, however, mainly acute driven, with a general lack of sufficient attention to patients’ chronic needs and adequate education of chronically ill patients in managing their conditions and protecting against further deterioration of their well-being. Patients’ visits to doctors are usually brief and characterized by uninformed passive interaction with unprepared healthcare professionals, resulting in frustrating and ineffective encounters [3, 4].

To meet the needs and protect the well-being of chronically ill patients, a patient-centered system of care delivery characterized by high-quality proactive care that is organized, structured, and planned through a focus on interactions between informed, activated patients, and proactive healthcare teams is needed. Thus, patients need to be *informed* (provided with sufficient information to become proactive partners and wise decision makers in their care delivery) and *activated* (by understanding the importance of information sharing and their roles in managing the illness). In addition, teams of healthcare professionals must be organized, trained, and equipped to conduct productive interactions, provide patient-centered care, and coproduce care delivery.

Traditionally, care delivery took a more paternalistic form in which relationships between patients and healthcare professionals were defined by norms of professional autonomy and role-based power, rather than shared decision making [7]. Currently, patient-centered care is advocated as the way to achieve such shared medical decision making [7, 8]. Research, however, showed that most patients do not feel that their level of participation in medical decision making is sufficient and several difficulties occur in the establishment of productive patient–professional interaction [9]. Patients as well as professionals are increasingly expected to possess the right communication skills [10, 11] which not all people have. Patients are also expected to be more assertive and involved to enable a patient-centered approach to medical decision making [12, 13]; not all patients are able to take this assertive and involved role in their care delivery. Furthermore, preferences in shared decision making are known to vary among patients. Professionals should, therefore, be more sensitive to patients’ individual preferences and regularly ask patients about their wish to be involved in the decision-making process [14]. This calls for a patient-centered approach with professionals performing their role in a less authoritarian manner [15]. Healthcare professionals should make decisions in accordance with patients’ preferences by letting patients share these preferences and facts about their situations [7], which is expected to lead to more productive patient–professional interaction and the coproduction of care delivery. Joint decision making and responsibility taking are achieved through open communication, cooperation, and respect for each other, with negotiation of treatment options to accomplish mutually defined goals. Productive interaction between patients and professionals may be recognized by accurate, frequent, and problem-solving communication that is supported by relationships based on shared goals and mutual respect. Gittell [16, 17] identified this concept as “relational coproduction,” which refers to the coproduction of care delivery through combined equal contributions of patients and their healthcare professionals. Rather than a situation in which healthcare professionals tell patients what they must do or which treatment they should receive, the coproduction of care involves productive interaction characterized by reciprocal interrelating between healthcare professionals and patients regarding what needs to be done (goal setting) and how best to do it (treatment choices). Productive interactions are based on high levels of shared goals, shared knowledge, and mutual respect that together foster attentiveness to the situation and to one another [17]. In contrast, poor interaction between patients and healthcare professionals may result in precarious patient care delivery and poor patient outcomes. Failure to communicate accurately and share knowledge, or differences in treatment goals between patients and healthcare professionals may lead to lack of respect and finger pointing, resulting in a lack of motivation in both parties.

Although interest in the examination of productive interactions or coproduction of care between chronically ill patients and their healthcare professionals is growing, this area of research is relatively new, with a preponderance of conceptual literature over empirical studies [18]. Empirical investigations of the quality of chronic care, productive patient–professional interaction, and their contributions to more favorable patient outcomes (e.g., the enhancement of chronically ill patients’ well-being) are lacking. General practitioners (GPs), who usually have longer histories with patients than, for example, physical therapists or dieticians, may more readily achieve productive interactions with patients [19]. Furthermore, O’Leary et al. [20] found that members of certain disciplines involved in patient care held discrepant views about their work that may result in differences in interactions. Thus, this study aimed to investigate the levels of interaction between patients and various types of healthcare professionals and examine the relationship between quality of chronic care delivery, productive patient–professional interaction, and chronically ill patients’ well-being.

## Methods

### Study design and participants

This study included patients participating in 18/22 Dutch disease management programs, characterized as collaborations between care sectors (e.g., between GPs and hospitals) or within primary care settings (e.g., among pharmacists, physiotherapists, dieticians, and social workers). Four disease management programs were excluded due to differences in the timing of questionnaire distribution (*n* = 1) and questionnaire content [to address specific mental health conditions (psychotic disorders, depression, and eating disorders); *n* = 3]. The disease management programs included in the study targeted patients with cardiovascular diseases (*n* = 9), chronic obstructive pulmonary disease (COPD; *n* = 4), heart failure (*n* = 1), comorbidity (*n* = 1), and diabetes (*n* = 3) [21]. The ethics committee of the Erasmus University Medical Center of Rotterdam approved the study, and all participants provided informed consent.

In 2011 (T1), we sent questionnaires to all 4,693 patients participating in the 18 disease management programs; 2,191 respondents completed the questionnaire (47 % response rate). One year later (2012; T2), we sent questionnaires to 4,350 patients still participating in the disease management programs; 1,722 respondents completed the questionnaire at this time point (40 % response rate). A total of 1,279 patients completed questionnaires at both time points (T1 and T2).

### Measures

Well-being was measured at T1 and T2 with the 15-item version of the Social Production Function Instrument for the Level of Well-being (SPF-IL) [22]. This scale measures levels of physical (comfort, stimulation) and social (behavioral confirmation, affection, status) well-being. Examples of questions are: “Do people pay attention to you?” (affection), “Do you feel useful to others?” (behavioral confirmation), “Are you known for the things you have accomplished?” (status), “In the past few months have you felt physically comfortable?” (comfort), and “Do you really enjoy your activities?” (stimulation). Responses are structured by a four-point scale ranging from never (1) to always (4), with higher mean scores indicating greater well-being. This instrument has been proven to be reliable for the assessment of well-being in older populations [23–25] and in the general population [22]. Cronbach’s alpha values of the SPF-IL at T1 and T2 were 0.85 and 0.87, respectively, indicating good reliability.

We assessed productive interactions among patients and (teams of) healthcare professionals using an adjusted version of the Relational Coordination instrument at T2. Although originally developed for the airline industry [26], this instrument has also been used in hospital [27–29], primary care [30, 31], and community care [32] settings. These studies investigated the quality of communication and relationships (i.e., relational coordination) among healthcare professionals and did not include patients. In our study, this instrument was used to measure patients’ perceptions of their interactions with healthcare professionals (i.e., relational coproduction [17]) involved in the disease management programs (GPs, practice nurses, dieticians, physical therapists, medical specialists, and nurses). It contained three items assessing the quality of communication with each individual healthcare professional (How frequently do you communicate with the following professionals? Do these professionals communicate accurately with you? When problems arise regarding the care do these professionals work with you to solve the problem?) and two items concerning relationship dimensions (Do these professionals share the same goals as you? and Do the professionals respect you?). Responses are structured by a four-point scale (not at all–sometimes–often–always). Cronbach’s alpha of the instrument was 0.95, indicating excellent reliability.

The 11-item Patient Assessment of Chronic Illness Care–Short version (PACIC-S) was used to assess patients’ perceptions of the quality of chronic care delivery at T2 [6, 33]. Examples of question are: ‘given choices on treatment to think about,’ ‘given a copy of my treatment plan,’ ‘encouraged to go to a specific group/class to help me cope with my chronic illness,’ and ‘asked how my chronic illness affects my life.’ While originally validated with a five-point scale, a four-point scale (ranging from 1 to 4, with higher scores indicating better perceptions of quality of care) was used to assess the quality of chronic care in 2012 in this study. Cronbach’s alpha of the PACIC-S was 0.88, indicating good reliability.

We also asked participants to provide information on background characteristics, such as age, gender, marital status, and education. Patients’ educational levels were characterized using six levels ranging from 1 [no school or primary education (≤7 years)] to 6 [university degree (≥18 years)]. We dichotomized this item into low (no school or primary education) and high (more than primary education) educational level.

### Statistical analyses

First, descriptive statistics were used to describe the study population and patients’ assessments of the quality of chronic care and interactions with healthcare professionals. Paired sample *t* tests were used to investigate differences in well-being over time (T1 vs. T2). Second, we employed correlation analyses—the Pearson or the Spearman Rho correlations if appropriate—to investigate associations among individual characteristics, quality of chronic care, productive interaction between patients and (teams of) healthcare professionals, and well-being. Third, we used a multilevel random-effects model to investigate the predictive roles of the quality of chronic care delivery and productive patient–professional interaction while controlling for patients’ well-being at T1, age, gender, educational level, and marital status. Results were considered statistically significant if two-sided *p* values were ≤0.05.

## Results

Table 1 displays the characteristics of the 1,279 patients who completed both questionnaires (at T1 and T2). About half (45 %) of the respondents were female, 38 % had a low educational level, and 31 % were single. Respondents’ mean age was 67.83 ± 10.02 (range 16–94) years. Among assessments of interactions with healthcare professionals, patients’ ratings of the quality of the relationship (shared goals 3.22 ± 0.87; mutual respect 3.49 ± 0.79) were higher than those of the quality of communication (frequent communication 2.20 ± 0.80; accurate communication 2.83 ± 0.98; problem-solving communication 3.02 ± 0.98). Chronically ill patients’ well-being improved slightly from 2.76 at T1 to 2.79 at T2 (*n* = 1,209; *p* ≤ 0.05).

**Table 1:** Descriptive statistics of patients participating in disease management programs in the Netherlands at T2

	Mean ± standard deviation (range) or percentage
Age (years)	67.62 ± 10.03 (16–94)
Gender (female)	45 %
Marital status (single)	31 %
Low educational level	38 %
Patients’ perceptions of quality of chronic care	2.13 ± 0.71 (1–4)
Well-being	2.79 ± 0.46 (1–5)
*Quality of communication*	
Frequent communication	2.20 ± 0.80 (1–4)
Accurate communication	2.83 ± 0.98 (1–4)
Problem-solving communication	3.02 ± 0.98 (1–4)
*Quality of relationship*	
Shared goals	3.22 ± 0.87 (1–4)
Mutual respect	3.49 ± 0.79 (1–4)
Overall interactions between patients and professionals	2.93 ± 0.73 (1–4)

Analyses included only respondents who filled in questionnaires at both T1 and T2 (*n* = 1,279)

Table 2 displays patients’ perceptions of their interactions with healthcare professionals in the context of the disease management programs. They reported a higher degree of interaction with GPs than with professionals in other disciplines.

**Table 2:** Patients’ rating of the productivity of interactions with healthcare professionals within the context of Dutch disease management programs

Interactions between patients and	Mean	Standard deviation
General practitioners	3.18	0.72
Practice assistants	2.77	1.04
Medical specialists	2.37	1.10
Nurses	1.75	1.04
Physical therapists	2.12	1.19
Dieticians	1.57	0.95

Analyses included only respondents who filled in questionnaires at both T1 and T2 (*n* = 1,279)

Associations among individual characteristics, quality of chronic care, productive interactions between patients and (teams of) healthcare professionals, and well-being are displayed in Table 3. The well-being of patients at T2 was strongly related to their well-being at T1 (*r* = 0.70), and weakly related to single status (*r* = –0.14), low educational level (*r* = –0.11), female gender (*r* = –0.11), quality of chronic care delivery (*r* = 0.12), and productivity of interactions with (teams of) healthcare professionals (*r* = 0.31; all *p* ≤ 0.001).

**Table 3:** Associations among individual characteristics, quality of chronic care, and productive interactions between patients and (teams of) healthcare professionals

	1	2	3	4	5	6	7
1. Well-being (T1)							
2. Age (T2)	0.05* (*n* = 1,239)						
3. Marital status (single) (T2)	–0.13*** (*n* = 1,228)	0.13*** (*n* = 1,238)					
4. Low educational level (T2)	–0.09*** (*n* = 1,192)	0.07* (*n* = 1,202)	0.07** (*n* = 1,198)				
5. Gender (female) (T2)	–0.09*** (*n* = 1,215)	–0.09*** (*n* = 1,227)	0.21*** (*n* = 1,223)	0.11*** (*n* = 1,186)			
6. Patients’ perceptions of quality of chronic care (T2)	0.09*** (*n* = 1,111)	–0.09*** (*n* = 1,220)	–0.05 (*n* = 1,108)	0.05* (*n* = 1,080)	–0.03 (*n* = 1,096)		
7. Productive interactions between patients and (teams of) healthcare professionals (T2)	0.20*** (*n* = 1,238)	–0.03 (*n* = 1,247)	–0.06* (*n* = 1,236)	–0.01 (*n* = 1,200)	0.01 (*n* = 1,224)	0.43*** (*n* = 1,130)	
8. Well-being (T2)	0.70*** (*n* = 1,219)	−0.01 (*n* = 1,225)	−0.14*** (*n* = 1,214)	−0.11*** (*n* = 1,182)	−0.11*** (*n* = 1,204)	0.12*** (*n* = 1,110)	0.31*** (*n* = 1,229)

Analyses included only respondents who filled in questionnaires at both T1 and T2 (*n* = 1,279). Results are based on the Spearman Rho correlations

*** *p* ≤ 0.001, ** *p* ≤ 0.01, * *p* ≤ 0.05 (two-tailed)

Table 4 displays the results of the multilevel analyses. After controlling for well-being at T1, age, marital status, educational level, and gender, the quality of chronic care clearly predicted the well-being of patients at T2 (*p* ≤ 0.01; Table 4, step 1). When productive interactions between patients and professionals at T2 were entered into the equation, it predicted the well-being of chronically ill patients (*p* ≤ 0.001) and mediated the relationship between the quality of chronic care and patients’ well-being. In step 2 of the model, the relationship between the quality of chronic care and patients’ well-being was no longer significant. More productive patient–professional interactions were related to better well-being at T2 (*B* = 0.11), assuming that all other factors in the model remained constant.

**Table 4:** Predictors of well-being at T2, as assessed by stepwise multilevel regression analyses (random intercepts model, *n* = 990)

	Step 1				Step 2			
	*β*	SE	*B*	SE	*β*	SE	*B*	SE
*Step 1*								
Constant	2.77	0.01	0.79	0.10	2.77	0.01	0.63	0.10
Well-being (T1)	0.32***	0.01	0.71***	0.03	0.31***	0.01	0.69***	0.02
Age (T2)	−0.00	0.01	−0.00	0.00	−0.01	0.01	−0.00	0.00
Marital status (single) (T2)	−0.02*	0.01	−0.04*	0.02	−0.02	0.01	−0.03	0.02
Low educational level (T2)	−0.02	0.01	−0.05	0.02	−0.02	0.01	−0.04	0.02
Gender (female) (T2)	−0.01	0.01	−0.03	0.02	−0.01	0.01	−0.03	0.02
Patients’ perceptions of quality of chronic care delivery (T2)	0.03**	0.01	0.05**	0.02	−0.00	0.01	−0.00	0.02
*Step 2*								
Productive interactions between patients and professionals (T2)					0.08***	0.01	0.11***	0.02

Multilevel analyses included only respondents who filled in questionnaires at both T1 and T2 (*n* = 1,279). Listwise deletion of missing cases resulted in the inclusion of 990 cases

*SE* standard error

*** *p* ≤ 0.001, ** *p* ≤ 0.01, * *p* ≤ 0.05 (two-tailed)

## Discussion and conclusions

### Discussion

This study aimed to (i) investigate interactions between patients and various healthcare professionals and (ii) determine the association between quality of chronic care, productive patient–professional interactions and chronically ill patients’ well-being. The results showed that chronically ill patients perceived interactions with GPs to be most productive, followed by those with practice assistants, medical specialists, physical therapists, nurses, and dieticians. They were especially satisfied with the quality of relationships with their healthcare professionals. Given that chronically ill patients usually have longer histories with their GPs and visit them more frequently in comparison with other care professionals, practice nurses and GPs may have had more opportunities to invest in good patient–professional relationships leading to more productive interactions.

The coproduction of care delivery is based on connections and productive interactions between patients and healthcare professionals, as well as the impact of these interactions on patient outcomes, such as the enhancement of their well-being. Our findings have clear implications for healthcare professionals: to foster productive patient–professional interactions, potential disease management collaborators should be selected for and trained in relational as well as functional competence. Relational competence includes the ability to see the larger picture, in our case to support all needs of chronically ill patients. It includes the ability to see patients’ perspectives, empathize with their situations, and respect their needs and choices [30]. Encouraging conversation and interactions between patients and healthcare professionals may require investing in time spent with patients. Although this approach may increase costs in the short term, it may produce a long-term return on investment exceeding the resources needed to make the change [18].

The results of this study also indicate that the quality of chronic care and productive patient–professional interactions were associated with patients’ well-being over time. Furthermore, productive interactions between patients and healthcare professionals mediated the relationship between patients’ perceptions of quality of care delivery and chronically ill patients’ well-being. Improvement of the quality of chronic care delivery should always be accompanied by investment in the quality of relationships and communication between patients and professionals. Many examples of suboptimal patient–professional communication were reported in a recent review [18], including professionals’ failure to create environments and relationships allowing for effective communication with patients, patients’ withholding of information from their healthcare professionals, and professionals’ failure to provide information about treatment and medication to patients in an understandable way. This review also noted suboptimal collaboration issues, including overly brief consultations with patients, frequent changing of healthcare professionals, patients’ failure to show up for scheduled appointments, professionals’ failure to ensure that patients understand treatments and choices about them, professionals’ inability to motivate patients, and an imbalance in decision making with pronounced skewing toward healthcare professionals [18]. These issues will lead to poor patient–professional interaction, instead of productive interaction characterized by shared knowledge, mutual respect, problem-solving communication, and accurate, understandable, and frequent communication with the same healthcare professional. To improve patient outcomes and ensure a more patient-centered approach, investment in high-quality chronic care delivery and relationships between healthcare professionals and patients is thus important.

This study was limited by the analysis of patients’ reports and perceptions only, with no examination of the effects of care quality and productive interaction on objective health outcomes. Further research is necessary to assess the effects of productive interaction on clinical outcomes. And although we did find a significant association, this effect was only small. Furthermore, dealing with patient–professional interaction only addresses some of the factors that contribute to patient-reported outcomes such as well-being. Other factors such as self-efficacy, social participation, and having a positive perspective on the future may also explain improved well-being of chronically ill patients. Finally, nonresponse bias at T1 and T2 may have affected our findings; patients with poor well-being are more likely not to have responded to the questionnaire at both time points.

The strengths of this study include the investigation of patients’ perceptions of quality of care, productive interactions, and their effects on well-being in diverse patient populations, including those with cardiovascular conditions, lung diseases (COPD), diabetes, heart failure, and comorbidity. Although all of these diseases have very specific or unique aspects, the quality of chronic care and productive patient–professional interactions are important for all populations of chronically ill patients. We performed additional analyses to investigate the influence of the type of chronic condition on productive patient–professional interactions. After controlling for the type of condition, the quality of chronic care (step 1) and productive patient–professional interaction (step 2) still predicted patients’ well-being (results not shown—available on request).

## Conclusion

We can conclude that productive patient–professional interactions are associated with chronically ill patients’ well-being over time and mediate the relationship between well-being and patients’ perceptions of quality of care. Improvement of the quality of chronic care delivery should always be accompanied by investment in the quality of relationships and communication between patients and professionals. To foster these productive interactions between patients and professionals, communication should be accurate, frequent, and aimed at solving problems. Quality of communication goes hand in hand with quality of the relationship, which should be respectful and based on shared goals.
